# Reply to Williams et al

**DOI:** 10.1093/ofid/ofae134

**Published:** 2024-03-08

**Authors:** M Hong Nguyen, Luis Ostrosky-Zeichner, Peter G Pappas, Thomas J Walsh, Joseph Bubalo, Barbara D Alexander, Marisa H Miceli, Jeanette Jiang, Yi Song, George R Thompson

**Affiliations:** Department of Medicine, University of Pittsburgh, Pittsburgh, Pennsylvania, USA; Department of Medicine, McGovern Medical School, Houston, Texas, USA; Department of Medicine, University of Alabama at Birmingham, Birmingham, Alabama, USA; Department of Medicine, Weill Cornell Medicine, Cornell University, New York, New York, USA; Office of the Director, Center for Innovative Therapeutics and Diagnostics, Richmond, Virginia, USA; Departments of Pharmacy and Medicine, Oregon Health and Science University Hospital and Clinics, Portland, Oregon, USA; Department of Medicine, Duke University, Durham, North Carolina, USA; Department of Medicine, University of Michigan, Ann Arbor, Michigan, USA; Department of Medical Affairs, Astellas Pharma Global Development Inc ., Northbrook, Illinois, USA; Department of Medical Affairs, Astellas Pharma Global Development Inc ., Northbrook, Illinois, USA; Department of Medicine, UC Davis Health, Sacramento, California, USA

To the Editor—We appreciate the opportunity to provide a response to Williams et al regarding our study published in *Open Forum Infectious Diseases* in 2023 [1].

In general, we agree with most of the comments raised by the authors and believe that they accurately illustrate a continuing problem of inadequate support for the use of mold-active triazoles (MATs) by insurance companies, plans, and/or pharmacy benefit managers directing care in non–clinically relevant ways, resulting in high out-of-pocket costs to patients who need life-saving antifungal therapy. As a result, access to MATs, drug prices, and payor coverage are still major problems in the United States. However, our study was not designed to address costs to patients, but instead to focus on epidemiological, mycological, safety, and outcome data in real-world patients who received MAT prophylaxis.

Moreover, there are several key differences between our study and that of Williams et al, which may be responsible for the differences observed. First, our study extracted data primarily from large academic centers, which may reflect care that is different to that of the community hospital investigated by Williams et al. Additionally, among the 3 MATs assessed in our study, only voriconazole was generically available during the study period, and the availability of isavuconazole and posaconazole may have impacted overall treatment costs. Since then, posaconazole has now also become generically available and there are programs to ameliorate the burden for those patients in whom the cost of MATs is prohibitive. With that said, we do acknowledge that posaconazole is still very expensive from retail outlets.

Notably, we consider the finding of Williams et al that almost half of their patients transitioned from MATs to non-MATs of particular interest to this therapy area. Use of non-MAT therapies (such as echinocandins, amphotericin B, or fluconazole) may well be an acceptable cost-effective alternative; however, in cases where alternative therapy is used, careful monitoring of signs and symptoms of invasive fungal disease is warranted, as evidenced by their case of breakthrough *Aspergillus* infection occurring during treatment with fluconazole.

## Note

***Potential conflicts of interest***. All authors: No reported conflicts.
